# BMI moderates the association between physical fitness and spherical equivalent in Chinese college students: a cross-sectional study

**DOI:** 10.3389/fpubh.2026.1937229

**Published:** 2026-09-24

**Authors:** Jiahuan He, Feng Li, Fengjie Gu, Yonghua Wang

**Affiliations:** 1 School of Medical Humanities and Management, Wenzhou Medical University, Wenzhou, Zhejiang, China; 2 Department of Sport Science, Wenzhou Medical University, Wenzhou, Zhejiang, China

**Keywords:** body mass index, effect modification, health disparities, myopia, physical fitness, spherical equivalent

## Abstract

**Background:**

Myopia has become a global public health crisis, with young adults representing a priority population for prevention. While physical fitness is recognized as a protective factor, whether this protection is uniformly distributed across different BMI subgroups remains unclear. This study examined the moderating role of BMI in the relationship between physical fitness and spherical equivalent (SE) among Chinese university students.

**Methods:**

A cross-sectional study was conducted with 300 college students (100 normal weight, 100 overweight, 100 obese) from Zhejiang Province, China. Physical fitness and SE were measured using standardized protocols. Multiple linear regression, adjusted for sex, was performed to test the moderating effect of BMI.

**Results:**

Physical fitness was independently associated with SE (Model 1: *β* = 0.042, *p* = 0.001), while BMI showed no main effect (Model 1: *p* = 0.766). A significant negative interaction emerged between fitness and BMI (Model 2: *β* = −0.009, 95% CI: −0.013 to −0.004, *p* < 0.001). Simple slope analysis (post-hoc analysis of Model 2) revealed that the protective association of fitness was stronger at low BMI (*β* = 0.071, *p* < 0.001), moderate at mean BMI (*β* = 0.032, *p* = 0.011), and absent at high BMI (*β* = −0.007, *p* = 0.523). Johnson-Neyman analysis (post-hoc analysis of Model 2) identified a threshold at BMI_c = 0.744 (raw BMI ≈ 26.45 kg/m^2^), after which the association was no longer significant.

**Conclusion:**

Physical fitness protects against myopia in college students, but this benefit is significantly diminished among those with higher BMI, revealing heterogeneity in intervention effectiveness across weight subgroups. From a public health perspective, this finding suggests that universal physical activity promotion alone may not confer equitable vision benefits across all BMI groups. Stratified intervention strategies—combining weight management with structured exercise for overweight/obese subgroups—are warranted to ensure equitable outcomes in population-level myopia prevention.

## 1 Introduction

Myopia has emerged as a predominant public health crisis worldwide, with its prevalence among children and young adults reaching unprecedented levels over recent decades. Projections estimate that nearly half of the global population will be affected by myopia by 2050, with high myopia affecting approximately one billion individuals (1). The escalating burden of myopia extends beyond individual health, profoundly impairing quality of life and academic performance, while pathological myopia substantially elevates the risk of irreversible visual impairments, including retinal detachment, glaucoma, and myopic maculopathy (2–4). These concerning trends have galvanized research efforts to identify modifiable protective factors and develop effective, scalable prevention strategies. From a public health standpoint, young adults in university settings represent a priority population for integrated chronic disease prevention: this life stage is characterized by the establishment of long-term lifestyle patterns, yet it coincides with dual epidemics of rising myopia prevalence and increasing obesity rates—two conditions that share modifiable behavioral determinants but are typically addressed through siloed intervention programs (5).

Among the myriad factors investigated, physical fitness has garnered substantial at tention. A growing body of evidence demonstrates that higher physical fitness levels are consistently associated with more favorable refractive status, positioning fitness enhancement as a promising strategy for myopia prevention and control (6–8). Concurrently, body mass index (BMI)—a core indicator of nutritional status and adiposity—has emerged as a factor of increasing interest in myopia research. Epidemiological evidence indicates that overweight and obese individuals face significantly elevated risks of myopia compared with their normal-weight counterparts, suggesting that obesity may constitute an independent risk factor for myopia (9, 10). However, the prevailing research paradigm has largely treated physical fitness and BMI as independent, parallel factors, examining their separate effects on myopia without testing their potential interactive effects (11, 12).

This gap raises a critical yet under-explored scientific question: Is the protective association of physical fitness against myopia uniformly distributed across different BMI levels? In other words, does excess body weight modify the strength of the fitness-myopia association? Empirical evidence addressing this question remains scarce, and the underlying mechanisms of their interplay are poorly understood. Consequently, most university health promotion programs continue to adopt a “one-size-fits-all” approach to myopia prevention—a strategy that may fail to target high-risk subgroups effectively and, from an equity perspective, may inadvertently perpetuate or even widen disparities in visual health outcomes across student populations (13). This concern is particularly salient given that the “one-size-fits-all” approach assumes uniform responsiveness to interventions, an assumption that our study directly challenges.

Furthermore, the majority of existing research has focused on children and adolescents, leaving university students—a population with relatively stabilized refractive development yet persistently high myopia prevalence and rising obesity rates—largely understudied (14, 15). University students represent a uniquely valuable population for public health investigation: they are at a critical life stage during which lifestyle patterns become established, yet they face compounding environmental pressures—including prolonged near-work, reduced physical activity, and dietary transitions—that may simultaneously influence both BMI and myopia risk. Understanding how these factors interact in this population is essential for designing effective, equity-oriented population health interventions.

To address these gaps, the present study was designed with two primary objectives. First, we aimed to determine whether physical fitness level is independently associated with spherical equivalent (SE) among Chinese university students, after controlling for sex. Second, we sought to examine the moderating role of BMI in the relationship between physical fitness and SE, testing the hypothesis that the protective association of physical fitness on myopia is attenuated among individuals with higher BMI. By elucidating whether and how BMI modifies the fitness-myopia association—specifically, by identifying a potential effect modification pattern that can inform risk stratification—this study aims to provide empirical evidence for the development of stratified, precision-oriented population health interventions that promote health equity across diverse university populations (16). From a public health practice perspective, the identification of differential responsiveness to physical activity by weight status has direct implications for the design of proportionate universalism strategies, wherein intervention intensity is matched to the level of need, thereby ensuring that the benefits of health promotion are equitably distributed across all body size groups.

## 2 Methods

### 2.1 Study design and participants

Participants were recruited from students undergoing the National Student Physical Fitness Standard Test at a university in China. Using BMI categories defined by Chinese criteria for adults (normal weight: 18.5 ≤ BMI < 24.0 kg/m^2^; overweight: 24.0 ≤ BMI < 28.0 kg/m^2^; obesity: BMI ≥ 28.0 kg/m^2^), we randomly selected 100 students from each category, yielding a total sample of 300 participants. By design, equal numbers of participants (*n* = 100) were sampled from each of the three BMI categories (normal weight, overweight, and obese). Therefore, the BMI distribution and the proportions of myopia reported in this study reflect the sampling scheme and do not represent population prevalence estimates. All proportions reported are specific to this study sample. All participants were aged 18 years or older and had a BMI ≥ 18.5 kg/m^2^. Exclusion criteria included: (1) history of refractive surgery (including femtosecond laser, LASIK, or ICL implantation); (2) current use of orthokeratology lenses; (3) congenital amblyopia; (4) history of strabismus surgery; or (5) hyperopia.

### 2.2 Measures

#### 2.2.1 Physical fitness

Physical fitness was assessed using a composite score derived from standardized field tests mandated by the National Student Physical Fitness Standard Test for Chinese university students. The test battery included standing long jump, sit-and-reach, 50-meter sprint, endurance run (1,000 m for males, 800 m for females), and muscle strength tests (pull-ups for males, 1-min sit-ups for females). The composite score (range: 0–100) was calculated by weighting and aggregating performance across these dimensions, providing a comprehensive indicator of an individual’s overall physical fitness.

For descriptive purposes only (Table 1), we categorized this continuous composite score into four grades according to national standards: Excellent (≥ 90), Good (80–89), Passing (60–79), and Failing (< 60). For all regression analyses (Table 2), the continuous composite score was entered as the independent variable to maximize statistical power and preserve the full range of variation. Prior to regression, this continuous score was mean-centered to facilitate interpretation of the interaction term and reduce multicollinearity.

**Table 1:** Comparison of physical fitness levels across BMI groups.

Variable	Normal weight (*n* = 100)	Overweight (*n* = 100)	Obese (*n* = 100)	*p*-value
Physical fitness score, mean ± SD	78.92 ± 11.38	63.70 ± 11.95	58.49 ± 11.14	<0.001
*Post-hoc:* normal vs. overweight				<0.001
*Post-hoc:* normal vs. obese				<0.001
*Post-hoc:* overweight vs. obese				0.002
Fitness rating, *n* (%)				
Excellent (≥90)	13 (13.0)	2 (2.0)	1 (1.0)	
Good (80–89)	42 (42.0)	15 (15.0)	11 (11.0)	
Passing (60–79)	35 (35.0)	25 (25.0)	30 (30.0)	
Failing (<60)	10 (10.0)	58 (58.0)	58 (58.0)	

BMI groups (Chinese adult criteria): normal weight (18.5–23.9 kg/m^2^), overweight (24.0–27.9 kg/m^2^), obesity (≥28.0 kg/m^2^). Fitness ratings (National Student Physical Fitness Standards): excellent (≥90), good (80–89), passing (60–79), failing (<60). *p*-values from post-hoc pairwise comparisons after one-way ANOVA.

**Table 2:** Multiple linear regression analysis predicting spherical equivalent (SE).

Variable	Model 1 (Main effects)		Model 2 (Interaction)	
	*β* (95% CI)	*p*-value	*β* (95% CI)	*p*-value
Physical fitness (centered)	0.042 (0.017, 0.067)	0.001	0.032 (0.008, 0.057)	0.011
BMI (centered)	−0.012 (−0.092, 0.068)	0.766	−0.032 (−0.110, 0.047)	0.426
Physical fitness × BMI	–	–	−0.009 (−0.013, −0.004)	<0.001
Sex (ref: male)	−1.099 (−1.694, −0.504)	<0.001	−0.867 (−1.460, −0.274)	0.004
Constant	−3.477 (−3.837, −3.116)	<0.001	−3.907 (−4.322, −3.492)	<0.001
Model fit				
*R^2^*	0.077		0.122	
Adjusted *R^2^*	0.068		0.110	
Δ*R^2^* (vs. Model 1)	–		0.045	
*F*-statistic (df)	8.24 (3296)	<0.001	10.21 (4295)	<0.001
VIF	1.09, 1.67		1.07, 1.73	
Cohen’s *f^2^*	–		0.051	

Model 1 includes main effects of physical fitness, BMI, and sex; Model 2 additionally includes the physical fitness × BMI interaction term. All continuous variables were mean-centered prior to entry. *β* coefficients are unstandardized; values in parentheses are 95% confidence intervals. Sex was coded with male as the reference group. Model 1: *F* (3, 296) = 8.24, *p* < 0.001; Model 2: *F* (4, 295) = 10.21, *p* < 0.001. Δ*R^2^* represents the increment in *R^2^* from Model 1 to Model 2. VIF values are presented as the range across all predictors within each model. Cohen’s *f^2^* for the interaction term [*f^2^* = Δ*R^2^*/(1−*R^2^*_full)] was 0.051, indicating a small-to-medium effect size. SE is expressed in diopters (D).

#### 2.2.2 Spherical equivalent

Spherical equivalent (SE) was measured using a Lenstar biometer (LS 900, Haag-Streit, Switzerland), which also provided measurements of axial length, anterior chamber depth, lens thickness, and corneal curvature. SE was calculated as spherical power plus half the cylindrical power (SE = sphere + ½ cylinder). Following established practice in the literature, only right-eye data were used in the primary analyses to avoid statistical dependence from interocular correlation and to maintain consistency with prior research. Myopia severity was classified according to the “Myopia Prevention and Control Guidelines” issued by the National Health Commission of China: mild myopia (−3.00 D < SE ≤ −0.50 D), moderate myopia (−6.00 D < SE ≤ −3.00 D), and high myopia (SE ≤ −6.00 D).

#### 2.2.3 Body mass index

Body mass index was calculated as weight in kilograms divided by the square of height in meters (kg/m^2^). Height and weight were measured using standardized protocols during the physical examination. Following validated cutoffs for Asian populations (17), participants were classified into four BMI categories: underweight (BMI < 18.5 kg/m^2^), normal weight (18.5 ≤ BMI < 24.0 kg/m^2^), overweight (24.0 ≤ BMI < 28.0 kg/m^2^), and obesity (BMI ≥ 28.0 kg/m^2^). However, as our inclusion criteria required BMI ≥ 18.5 kg/m^2^, the underweight category was not applicable to this study sample. Therefore, participants were classified into three BMI categories: normal weight (18.5 ≤ BMI < 24.0 kg/m^2^), overweight (24.0 ≤ BMI < 28.0 kg/m^2^), and obesity (BMI ≥ 28.0 kg/m^2^).

### 2.3 Statistical analysis

All analyses were performed using Stata 17.0 (StataCorp, College Station, TX, USA). Continuous variables are presented as mean ± standard deviation (SD), and categorical variables as frequencies with percentages. Group comparisons were conducted using independent-samples *t*-tests for two-group comparisons and one-way analysis of variance (ANOVA) for comparisons involving three or more groups.

Prior to regression analyses, both physical fitness scores and BMI were mean-centered to reduce multicollinearity and facilitate interpretation of interaction terms. The categorical fitness grades (Excellent/Good/Passing/Failing) were used exclusively for descriptive comparisons in Table 1. Multiple linear regression models were constructed to examine the association between physical fitness and SE, with BMI as a moderator. The interaction term (physical fitness × BMI) was entered into the model to test for effect modification. All models were adjusted for sex. Axial length was not included as a covariate in the main models to avoid over-adjustment, as it lies on the causal pathway between physical activity and refractive status; sensitivity analyses adjusting for axial length are presented in the Supplementary Material.

Multicollinearity was assessed using variance inflation factors (VIF), with values below 5 considered indicative of no substantial collinearity. To interpret significant interactions, simple slope analysis was conducted to estimate the conditional effects of physical fitness on SE at low (mean – 1 SD), average (mean), and high (mean + 1 SD) levels of BMI. In addition, Johnson-Neyman (J-N) analysis was performed to identify the precise BMI threshold at which the conditional effect of physical fitness on SE transitioned from statistically significant to non-significant (*α* = 0.05). To further characterize the nature of the interaction, stratified regression analyses were conducted separately within each BMI category (normal weight, overweight, obese), with each stratum-specific model regressing SE on physical fitness and sex.

Effect sizes for the interaction were quantified using Δ*R^2^* (the increment in *R^2^* from the main-effects model to the interaction model) and Cohen’s *f^2^*, calculated as *f^2^* = Δ*R^2^*/(1−*R^2^*_full). Post-hoc power analysis for the interaction term was performed based on the observed effect size (*f^2^*), with power estimated using the non-central F distribution (*α* = 0.05, *N* = 300).

Results are reported with unstandardized regression coefficients (*β*), 95% confidence intervals (CIs), and *p*-values. Statistical significance was set at a two-sided *α* = 0.05 for all tests.

## 3 Results

### 3.1 Basic characteristics of study participants

As shown in Table 3, this study included 300 college students, comprising 184 males and 116 females. Significant gender differences were observed in BMI distribution, spherical equivalent (SE) and physical fitness scores (all *p* < 0.05). Males presented a higher average BMI and were overrepresented in the overweight and obese groups, with 71% of obese participants being male. By contrast, females had worse SE values and a higher proportion of myopic participants. Notably, females achieved higher physical fitness scores than males. These gender-related disparities suggest that sex is an important confounding variable, which was adjusted for in subsequent regression analyses.

**Table 3:** Basic characteristics of study participants by gender.

Characteristic	Total (*N* = 300)	Male (*n* = 184)	Female (*n* = 116)	*p*-value
BMI (kg/m^2^), mean ± SD	25.71 ± 4.41	26.41 ± 4.47	24.59 ± 4.08	0.004
BMI category, *n* (%)				<0.001
Normal weight	100 (33.3)	52 (28.3)	48 (41.4)	
Overweight	100 (33.3)	71 (38.6)	29 (25.0)	
Obese	100 (33.3)	61 (33.2)	39 (33.6)	
Spherical equivalent (D), mean ± SD	−3.90 ± 2.53	−3.62 ± 2.46	−4.35 ± 2.48	0.015
Physical fitness score, mean ± SD	67.03 ± 14.37	63.79 ± 14.57	72.18 ± 12.49	<0.001

Data are presented as mean ± standard deviation or n (%). SE = Spherical Equivalent. BMI groups were defined according to Chinese criteria for adults: Normal weight (18.5 ≤ BMI < 24.0 kg/m^2^), Overweight (24.0 ≤ BMI < 28.0 kg/m^2^), Obesity (BMI ≥ 28.0 kg/m^2^). *p*-values are derived from independent samples *t*-tests (for continuous variables) or chi-square tests (for categorical variables).

### 3.2 Comparison of myopia proportion across BMI groups

As shown in Table 4, the overall proportion of myopic participants in our sample was 86.67%. Significant differences in SE were detected across the three BMI groups (*p* < 0.001). Post-hoc tests showed that students with normal weight had significantly better refractive status than those in the overweight and obese groups. However, the overweight group had worse mean SE (−4.43 D) than the obese group (−4.12 D), with no statistically significant difference (*p* = 0.366). In addition, the proportion of non-myopic individuals decreased gradually with the rise of BMI. Students with overweight or obesity constituted the main high-risk subgroup for moderate and high myopia, reflecting obvious visual health disparities across weight categories.

**Table 4:** Comparison of myopia proportion and severity across BMI groups.

Variable	Normal weight (*n* = 100)	Overweight (*n* = 100)	Obese (*n* = 100)	*p*-value
Spherical equivalent (D), mean ± SD	−3.16 ± 2.55	−4.43 ± 2.55	−4.12 ± 2.33	<0.001
*Post-hoc:* normal vs. overweight				<0.001
*Post-hoc:* normal vs. obese				0.006
*Post-hoc:* overweight vs. obese				0.366
Myopia severity, *n* (%)				
No myopia (SE > −0.50 D)	19 (19.0)	12 (12.0)	9 (9.0)	
Mild (−3.00 D < SE ≤ −0.50 D)	38 (38.0)	13 (13.0)	19 (19.0)	
Moderate (−6.00 D < SE ≤ −3.00 D)	27 (27.0)	38 (38.0)	50 (50.0)	
Severe (SE ≤ −6.00 D)	16 (16.0)	37 (37.0)	22 (22.0)	

BMI groups were defined as: Normal weight (18.5 ≤ BMI < 24.0 kg/m^2^), Overweight (24.0 ≤ BMI < 28.0 kg/m^2^), and Obesity (BMI ≥ 28.0 kg/m^2^) based on Chinese criteria for adults. SE values are presented as mean ± standard deviation in diopters (D). Myopia severity was classified as: No myopia (SE > − 0.50 D), Mild (−3.00 D < SE ≤ − 0.50 D), Moderate (−6.00 D < SE ≤ − 3.00 D), and Severe (SE ≤ − 6.00 D). *p*-values are derived from post-hoc pairwise comparisons following a significant one-way ANOVA. Due to the equal-size sampling design (*n* = 100 per BMI group), the proportions shown in this table reflect the study sample and should not be interpreted as population prevalence estimates.

### 3.3 Comparison of physical fitness level across BMI groups

As shown in Table 1, physical fitness scores showed a clear gradient trend across BMI groups (*p* < 0.001). The normal weight group had the highest fitness level, followed by the overweight group, while the obese group obtained the lowest scores. Post-hoc pairwise comparisons confirmed significant differences between every two groups. In terms of fitness grading, over 90% of students with substandard physical fitness belonged to the overweight and obese groups. This indicates that elevated BMI is closely linked to poor physical fitness among college students, and the coexistence of high BMI and low fitness forms a prominent dual health risk.

### 3.4 Correlation analysis

Table 5 presents the bivariate correlations among key study variables. Physical fitness score was significantly positively correlated with SE (*r* = 0.189, *p* = 0.002), while BMI (*r* = −0.123, *p* = 0.035) and axial length (*r* = −0.766, *p* < 0.001) were significantly negatively correlated with SE. Axial length exhibited the strongest correlation with SE. Due to this high correlation and its role in the causal pathway, it was not included as a covariate in the main regression models. Sex was also significantly correlated with SE (*r* = −0.140, *p* = 0.016).

**Table 5:** Bivariate correlations among key variables.

Variable	SE	Physical fitness	BMI	Axial length	Sex
SE	1.000				
Physical fitness	0.189^**^	1.000			
BMI	−0.123^*^	−0.610^***^	1.000		
Axial length	−0.766^***^	−0.200^**^	0.174^**^	1.000	
Sex	−0.140^*^	0.285^***^	−0.203^***^	−0.178^**^	1.000

SE = spherical equivalent (in diopters). Physical fitness = continuous composite score (range: 0–100), mean-centered prior to regression analysis. BMI = body mass index (kg/m^2^). Axial length (mm). Sex: 1 = male, 2 = female. *p* < 0.05, ^**^p < 0.01, ^***^p < 0.001 (two-tailed).

### 3.5 Regression analysis and moderation effect

Multiple linear regression was performed to examine the association between physical fitness and SE, with BMI as a moderator, adjusting for sex. Table 2 presents the regression results. In Model 1 (main effects only), physical fitness was significantly associated with SE (*β* = 0.042, 95% CI: 0.017 to 0.067, *p* = 0.001), while BMI showed no significant main effect (*β* = −0.012, 95% CI: −0.092 to 0.068, *p* = 0.766). Sex was also a significant predictor (*β* = −1.099, 95% CI: −1.694 to −0.504, *p* < 0.001). The model explained 7.7% of the variance in SE (adjusted *R^2^* = 0.068). Multicollinearity was assessed using variance inflation factors (VIF); all VIF values ranged from 1.09 to 1.67 (mean VIF = 1.45), indicating no substantial multicollinearity.

In Model 2 (with interaction term), the interaction between physical fitness and BMI was statistically significant (*β* = −0.009, 95% CI:–0.013 to −0.004, *p* < 0.001), indicating that BMI significantly moderated the relationship between physical fitness and SE. Multicollinearity was assessed using variance inflation factors (VIF); all VIF values ranged from 1.07 to 1.73 (mean VIF = 1.39), indicating no substantial multicollinearity. The model explained 12.2% of the variance in SE (adjusted *R^2^* = 0.110), with the interaction term accounting for an additional 4.5% of the variance (Δ*R^2^* = 0.045, *p* < 0.001, Cohen‘s *f^2^* = 0.051). Sex remained a significant predictor in Model 2 (*β* = −0.867, 95% CI: −1.460 to −0.274, *p* = 0.004).

Simple slope analysis was conducted to interpret the significant interaction (Figure 1). The protective association of physical fitness on SE was strongest among individuals with low BMI (*β* = 0.071, *p* < 0.001), moderate at mean BMI (*β* = 0.032, *p* = 0.011), and non-significant at high BMI (*β* = −0.007, *p* = 0.523). These results indicate that the beneficial association of physical fitness with SE progressively diminishes as BMI increases. Johnson-Neyman analysis further identified a threshold at BMI_c = 0.744 (corresponding to raw BMI ≈ 26.45 kg/m^2^), beyond which the protective association was no longer statistically significant (Figure 2).

**Figure 1:**
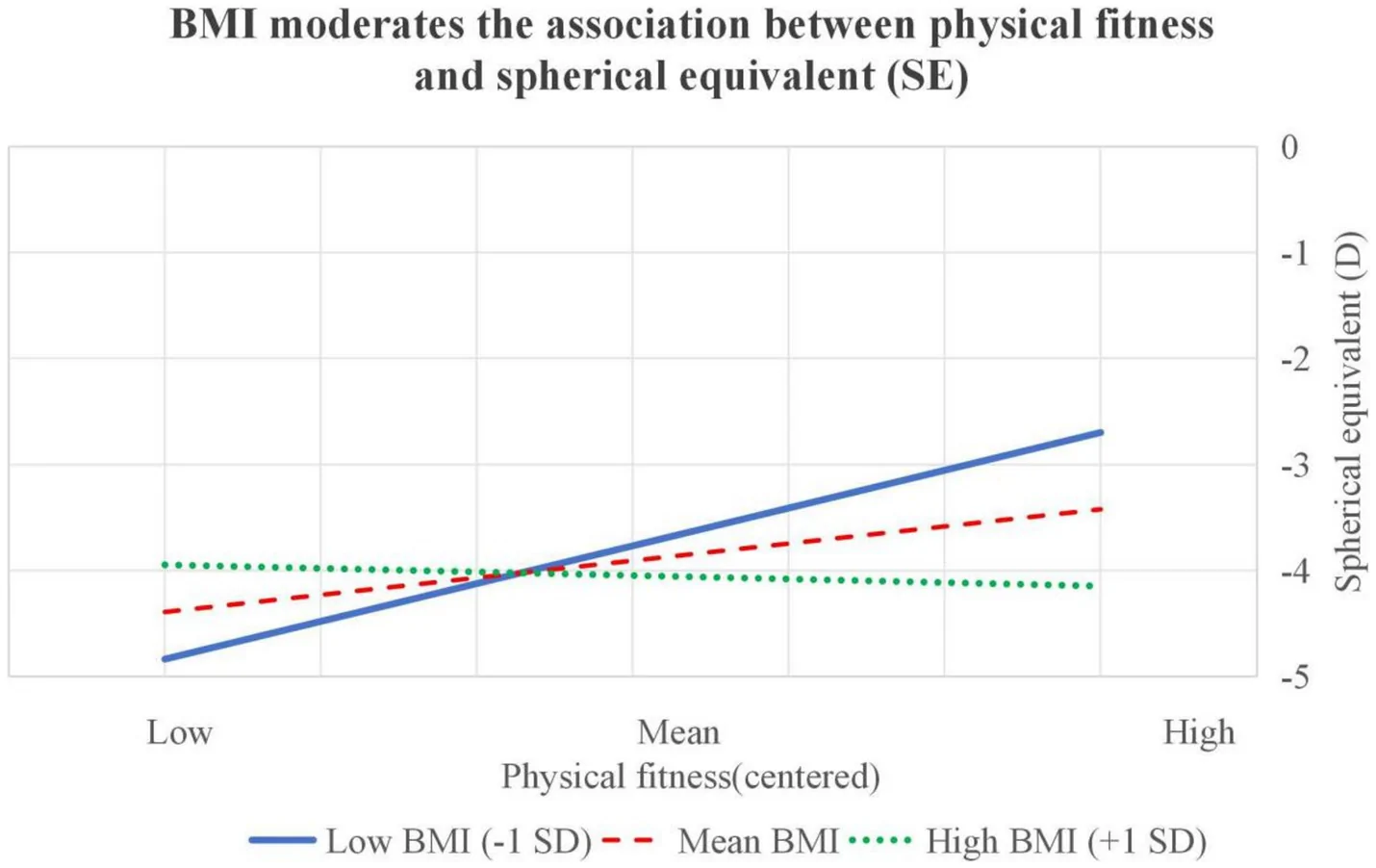
Simple slopes of fitness-SE association by BMI. Lines represent predicted SE values across centered physical fitness scores at three BMI levels: low (−1 SD), mean, and high (+1 SD). The model was adjusted for sex. Simple slope analysis revealed that the association between physical fitness and SE was strongest at low BMI (*β* = 0.071, *p* < 0.001), moderate at mean BMI (*β* = 0.032, *p* = 0.011), and non-significant at high BMI (*β* = −0.007, *p* > 0.05). The significant fitness × BMI interaction (*β* = −0.009, *p* < 0.001) indicates that the protective association of physical fitness against myopia is progressively attenuated with increasing BMI. BMI, body mass index; SE, spherical equivalent; SD, standard deviation.

**Figure 2:**
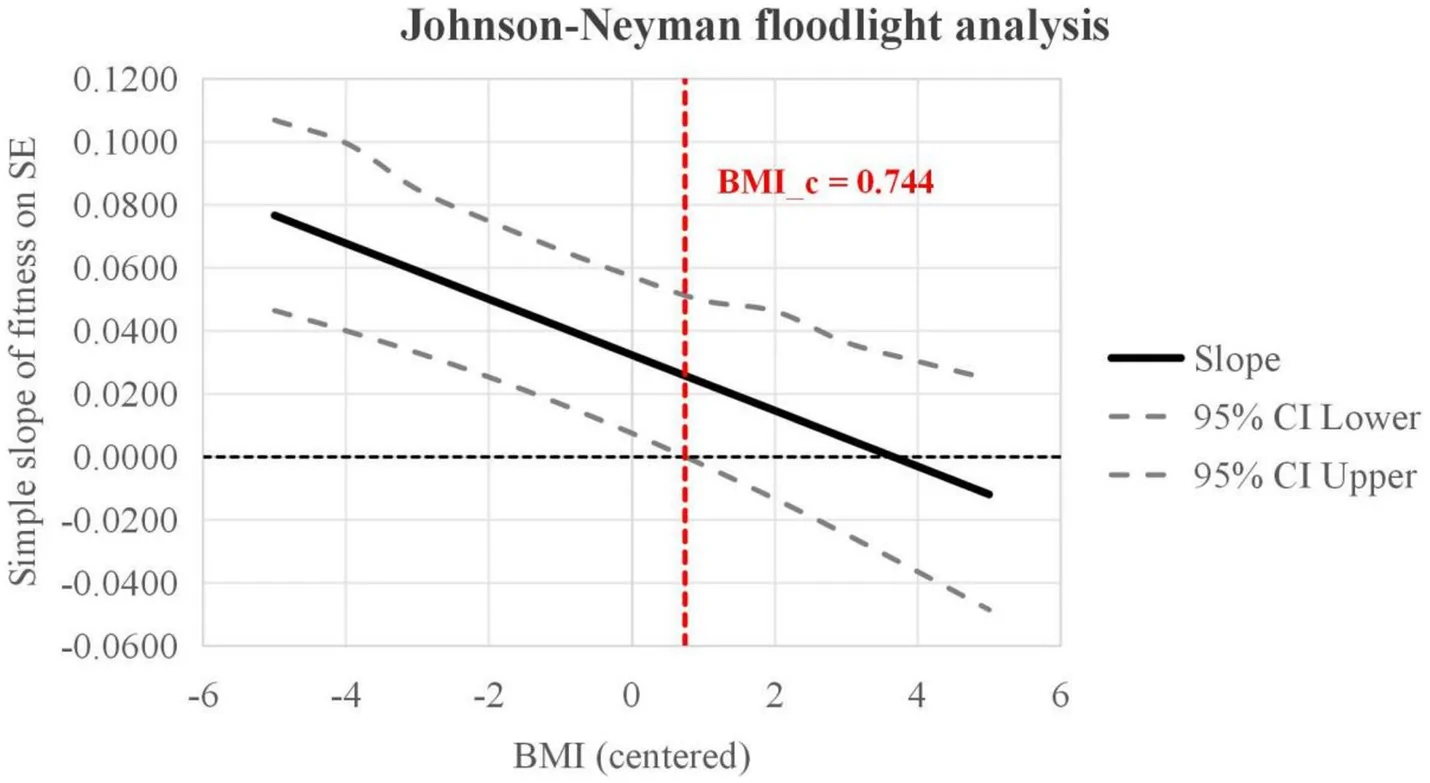
Johnson-Neyman floodlight analysis. The solid line represents the simple slope of physical fitness on spherical equivalent (SE) as a function of centered BMI (BMI_c). Dashed lines represent the 95% confidence bands. The horizontal dashed line at zero indicates the null effect (i.e., no association between fitness and SE). The vertical dashed line marks the Johnson-Neyman threshold at BMI_c = 0.744 (corresponding to raw BMI ≈ 26.45 kg/m^2^). The association was statistically significant when BMI_c was below this threshold (*p* < 0.05) and non-significant when above (*p* > 0.05).

To further examine the nature of this moderating effect, we performed stratified regression analyses by BMI category (normal, overweight, obese), estimating the fitness-SE association separately within each stratum, adjusted for sex (Supplementary Table S2). A clear descending gradient was observed: the fitness coefficient was largest in the normal-weight group (*β* = 0.088, 95% CI: 0.047 to 0.130, *p* < 0.001), markedly attenuated in the overweight group (*β* = 0.014, 95% CI: −0.024 to 0.053, *p* = 0.455), and completely nullified in the obese group (*β* = −0.035, 95% CI: −0.086 to 0.015, *p* = 0.170). This gradient parallels the J-N threshold, further supporting the interpretation that the protective association of physical fitness is progressively attenuated with increasing BMI and becomes negligible beyond a critical BMI value. Sensitivity analyses adjusting for axial length, presented in Supplementary Table S1, showed consistent patterns (interaction *β* = −0.003, 95% CI: −0.006 to −0.000, *p* = 0.046), supporting the robustness of these findings.

## 4 Discussion

This population-based cross-sectional analysis of Chinese college students yielded three robust findings that advance understanding of the intertwined associations between physical fitness, body mass index, and myopia, with direct implications for public health practice. First, higher physical fitness was linked to less myopic refraction after controlling for sex, pointing toward a potential protective role of regular physical activity in young adults‘visual health. Second, while BMI exerted no direct main effect on spherical equivalent, a statistically significant negative moderating interaction was identified between physical fitness and BMI (*β* = −0.009, 95% CI:–0.013 to −0.004, *p* < 0.001). Simple slope analysis further revealed that the protective association of physical fitness with SE progressively diminished as BMI increased: the association was strongest in the low-BMI group (*β* = 0.071, *p* < 0.001), moderate at mean BMI (*β* = 0.032, *p* = 0.011), and absent in the high-BMI group (*β* = −0.007, *p* = 0.523). Johnson-Neyman analysis further identified a threshold at BMI_c = 0.744 (corresponding to raw BMI ≈ 26.45 kg/m^2^), beyond which the protective association was no longer statistically significant. Third, pronounced visual health disparities were observed across weight subgroups: normal-weight students maintained superior refractive status and higher fitness levels, whereas overweight and obese individuals constituted a high-risk cluster characterized by compound burdens of poor physical fitness and more severe myopia. Stratified regression analyses confirmed a clear descending gradient (0.088 → 0.014 → –0.035), further supporting the moderating role of BMI. Collectively, these results indicate that the visual health gains conferred by physical activity are not uniformly distributed across all weight groups, revealing a heterogeneity in intervention effectiveness that carries direct implications for the design of population-level myopia prevention strategies.

### 4.1 Comparison with existing literature and theoretical innovations

The positive independent association between physical fitness and favourable refractive status observed in this study aligns with a substantial body of epidemiological evidence conducted among child and adolescent populations (18–20). A widely proposed mechanism for this association is increased outdoor light exposure, which facilitates retinal dopamine release and inhibits axial elongation (21, 22), while regular physical activity may also enhances ocular blood circulation and metabolic function, supporting healthy scleral remodeling and retinal health (23, 24). While these pathways are not mutually exclusive, it is important to note that physical fitness and outdoor time are related but not interchangeable—fitness may partly reflect an active lifestyle that includes outdoor activities, but it does not directly measure light exposure. Since we did not directly measure outdoor time or near-work intensity in this study, we cannot determine the relative contribution of these pathways. However, the observed progressive attenuation of the fitness-SE association across BMI levels suggests that adiposity status may influence the translation of physical activity into visual health benefit, regardless of the specific pathway.

Therefore, the present study extends this established evidence base in two dimensions that are particularly relevant to public health. First, most prior investigations have treated physical fitness and BMI as independent parallel predictors of myopia, neglecting their interactive synergies and failing to examine whether weight status modifies the strength of fitness-related protection (25). By systematically testing BMI’s moderating effect within a young adult college sample, this study provides new empirical evidence for this under-researched area and offers an explanatory framework that may help reconcile the heterogeneous effect sizes reported across previous population studies. Second, the majority of existing research has focused on primary and secondary school students, whose ocular structures remain developmentally plastic. College students represent a distinct and understudied cohort characterized by stabilized ocular development, sustained high academic load, and rapidly increasing overweight and obesity prevalence; our subgroup-stratified evidence supplements empirical data specific to young adult campus populations, thereby contributing to the generalizability of fitness-myopia research across the full youth life course.

In the present study, the main effect of BMI on spherical equivalent did not reach statistical significance—an observation that is not entirely consistent with findings from some large-scale population studies. However, the primary contribution of this study lies not in testing the main effect of BMI, but in revealing its moderating role (*β* = −0.009, *p* < 0.001) in the fitness-myopia relationship—specifically, that the protective association of physical fitness on myopia is significantly attenuated among individuals with higher BMI. The robustness of this moderating pattern was examined through sensitivity analyses adjusting for axial length (Supplementary Table S1). The primary model (without axial length) yielded an interaction estimate of *β* = −0.009 (95% CI: −0.013 to −0.004, *p* < 0.001). When axial length was included as a covariate, the interaction estimate was attenuated but remained directionally consistent (*β* = −0.003, 95% CI: −0.006 to −0.000, *p* = 0.046). Taken together, these primary and sensitivity estimates suggest that the true interaction effect lies between the two estimates. Moreover, a noteworthy observation in our data is a non-monotonic descriptive pattern: the overweight group showed a mean SE of −4.43 D versus −4.12 D in the obese group, although this group difference was not statistically significant (*p* = 0.366). Nominally, this seems at odds with a simple linear expectation that higher BMI would correspond to more severe myopia. However, this observation can be contextualized with the Johnson-Neyman threshold estimated in our model. The critical value on the centered BMI scale was 0.744, corresponding to a raw BMI of approximately 26.45 kg/m^2^. The overweight group (mean BMI ≈ 25.7 kg/m^2^) lies close to this critical boundary, representing a potential transition zone where the protective association of physical fitness for SE rapidly diminishes but has not yet fully disappeared—accompanied by relatively large SE variability in this sample (SD = 2.55). By contrast, the obese group (BMI ≥ 28 kg/m^2^) lies above this threshold, where the fitness-related protective association is no longer statistically detectable, with comparatively moderate SE dispersion (SD = 2.33). This interpretive framework receives further support from the gradient observed in stratified regression: the fitness-SE association was robust in the normal-weight group (*β* = 0.088), markedly attenuated in the overweight group (*β* = 0.014), and close to zero in the obese group (*β* = −0.035). Coefficient estimates within the obese stratum should be interpreted cautiously due to extremely limited representation of participants with excellent physical fitness.

Importantly, this descriptive non-monotonic pattern in crude BMI-SE means does not constitute evidence against BMI’s moderating role. Instead, it refines our interpretation of this moderation: rather than a simple linear attenuation across BMI values, the moderating effect exhibits threshold-dependent characteristics. The overall interaction term (*p* < 0.001) captures the general trend of effect attenuation across the BMI continuum, while the J-N threshold marks the raw-BMI cutoff beyond which the conditional protective association of fitness is no longer statistically significant. Thus, the observed non-monotonic descriptive pattern does not undermine our core conclusion; rather, it illustrates the non-linear, threshold-dependent nature of BMI moderation.

This finding suggests that even if the direct association between BMI and refractive status varies across studies, BMI’s role as an effect modifier carries independent empirical value: it indicates that population-level intervention strategies should be differentially designed according to weight status, rather than adopting uniform physical activity promotion approaches. From a public health perspective, identifying “who benefits from physical activity and who benefits less” is as important as identifying “who is at risk”.

### 4.2 Mechanistic interpretation and the role of competing exposures

The blunting effect of elevated BMI on fitness-mediated myopia protection can be understood through an integrated framework encompassing biological and behavioural pathways. From a behavioural perspective, overweight and obese college students frequently engage in sedentary lifestyles, avoid compulsory physical training, and exhibit lower voluntary outdoor activity participation, creating a vicious cycle of high adiposity, insufficient light exposure, and accelerated myopia progression (26). However, it must be emphasized that physical fitness is not synonymous with outdoor time; while fitness may correlate with active lifestyles, it does not directly measure light exposure, and the protective association of fitness could also arise from other mechanisms (e.g., improved vascular function, reduced inflammation). The overall pattern observed in our data—a progressive attenuation of the fitness-SE association from normal-weight (*β* = 0.088) to overweight (*β* = 0.014) to obese (*β* = −0.035) individuals—suggests that the “conversion efficiency” of physical activity into visual health benefit is substantially reduced among students with higher BMI.

From a biological perspective, chronic low-grade inflammation and metabolic dysregulation accompanying excess adipose tissue may interfere with scleral remodelling and ocular microcirculation (27, 28), partially counteracting the ocular protective association induced by regular physical activity (29, 30). However, this biological pathway serves as a supplementary rather than primary explanation; the core driver of attenuated fitness protection likely lies in a combination of socially shaped behavioural disparities across BMI subgroups and the physiological consequences of excess adiposity, rather than purely ocular structural changes. This layered interpretation—acknowledging both biological and social determinants—aligns with a population health perspective that recognizes the multifactorial nature of chronic disease risk.

Importantly, the behavioural interpretation above remains speculative in the absence of direct measures of outdoor time and near-work intensity—two factors unequivocally established as major determinants of refractive progression in student populations. Recent evidence demonstrates that intensive near-work demands accelerate myopic progression significantly (*β* = −0.161 D/year; 95% CI: −0.219 to −0.103; *p* < 0.001) (31), and prolonged electronic device use is associated with increased myopia risk (OR = 1.49; 95% CI: 1.00 to 2.23; *p* = 0.049) (32). Placing our results within this framework, it is plausible that heavier academic schedules simultaneously reduce physical activity, increase BMI, and promote myopia—a pathway that could confound or compete with the fitness–myopia association we observed. In this scenario, the observed fitness–SE association could be partially driven by reduced near-work time among more active students rather than by physical activity per se. Conversely, fitness may exert a protective association independent of near-work through vascular, inflammatory, or metabolic pathways. Without direct measurement of these behavioural factors, we cannot fully disentangle the specific contribution of fitness from that of correlated lifestyle or educational exposures.

Despite these limitations, the overall pattern of our findings—the stratified gradient across the full sample and the significant interaction—remains robust. However, given the extremely limited number of participants with both high fitness and obesity in our sample (only 1% of the obese group achieved an “excellent” fitness rating), we caution against over-interpreting the characteristics of this specific subgroup. Future studies integrating detailed measures of both physical activity patterns and near-work behaviours, in larger samples with balanced representation across fitness–BMI combinations, are warranted to disentangle these intertwined pathways.

### 4.3 Public health implications and implications for population intervention design

The moderating relationship and visual health disparities identified in this study carry substantial implications for population health practice and policy, extending beyond campus settings to inform national youth chronic disease prevention strategies. These findings challenge a fundamental assumption underlying many current public health interventions: that uniform, population-wide recommendations confer equal benefits across all subgroups. The observed BMI-dependent attenuation of fitness-related myopia protection reveals heterogeneity in intervention effectiveness that has direct implications for the design of precision public health strategies.

Importantly, as a cross-sectional study, this research does not aim to establish causality but rather to identify a previously unrecognized effect modification pattern—the differential responsiveness to physical activity by BMI status—that has critical implications for population intervention design. In public health practice, understanding who benefits and who does not from existing interventions is as essential as establishing causal effects. Our simple slope analysis revealed that the protective association of physical fitness on myopia was stronger among normal-weight individuals and substantially attenuated among overweight and obese individuals. Our simple slope analysis revealed that the protective association of physical fitness with myopia was strongest at low BMI (mean −1 SD; *β* = 0.071, *p* < 0.001), moderate at mean BMI (*β* = 0.032, *p* = 0.011), and absent at high BMI (mean +1 SD; *β* = −0.007, *p* = 0.523). Johnson-Neyman analysis further identified a threshold at BMI_c = 0.744 (corresponding to raw BMI ≈ 26.45 kg/m^2^), beyond which the protective association was no longer statistically significant. Stratified regression confirmed a clear descending gradient (0.088 → 0.014 → −0.035), supporting the moderating role of BMI across the full weight spectrum.

Using the Chinese BMI classification criteria for adults (overweight: 24.0–27.9 kg/m^2^; obesity: ≥28.0 kg/m^2^) as a reference, this attenuation pattern was consistently observed across overweight and obese subgroups, suggesting that BMI categories can serve as a preliminary basis for risk stratification in population-level myopia prevention—a practical contribution that does not require causal inference to be actionable. Notably, the Johnson-Neyman threshold identified in our study (approximately 26.45 kg/m^2^) falls within the overweight range defined by Chinese criteria, providing a statistically derived reference point that complements the existing clinical cutoff. Furthermore, although the interaction effect accounted for a modest proportion of variance (Δ*R^2^* = 0.045), in the context of complex multifactorial outcomes—where individual behavioral and environmental factors typically explain small proportions of variance—this effect carries substantive meaning; even a modest effect, when aggregated across populations with high myopia burden, could translate into considerable health disparities. In our sample, 86.7% of participants were myopic, illustrating the high disease burden in this population.

#### 4.3.1 Recommendation 1: risk-stratified population health protocols

Public health agencies and educational institutions should move beyond “one-size-fits-all” physical activity prescriptions. We recommend establishing simple risk stratification matrices that cross-reference fitness, BMI, and vision screening data to identify high-risk subgroups (e.g., “low fitness + high BMI”). For normal-weight populations, routine physical activity promotion suffices; for overweight/obese populations, enhanced intervention packages—combining structured weight management, accessible exercise programmes, and nutrition guidance—should be prioritized. This proportionate universalism approach ensures that intervention intensity matches the level of need, a core principle of effective and equitable population health strategy. The Johnson-Neyman threshold identified in our study (BMI ≈ 26.45 kg/m^2^) provides a statistically derived reference point for such stratification, which falls within the overweight range defined by Chinese criteria (BMI ≥ 24 kg/m^2^), offering a practical, empirically grounded basis that can be readily implemented in routine public health screening.

#### 4.3.2 Recommendation 2: integrated surveillance systems for chronic disease prevention

From a public health systems perspective, the integration of multiple student health monitoring systems—physical fitness testing, BMI screening, and vision assessment—should be promoted at institutional and regional levels. This integrated data infrastructure serves dual functions: (1) enabling early warning and precision targeting of high-risk individuals; and (2) facilitating continuous equity monitoring—tracking whether improvements in visual health are equitably distributed across weight subgroups over time. Without such monitoring, even well-intentioned interventions may inadvertently reinforce existing disparities by disproportionately benefiting those who are already advantaged. This represents a governance innovation that embeds health equity considerations into routine public health operations.

#### 4.3.3 Recommendation 3: health equity considerations for stratified myopia prevention

The clear descending gradient in the fitness-SE association—from normal-weight (*β* = 0.088) to overweight (*β* = 0.014) to obese (*β* = −0.035)—carries direct implications for health equity in myopia prevention. This gradient pattern indicates that the conversion “efficiency” of physical activity into visual health benefit progressively declines with increasing BMI, and the Johnson-Neyman threshold (≈26.45 kg/m^2^) suggests that beyond this critical value, the protective association is no longer statistically significant. This finding challenges the implicit assumption that adoption of health-promoting behaviours yields comparable benefits across all individuals. From a public health equity perspective, this pattern reveals differential “conversion efficiency”—the capacity to translate physical activity into health outcomes is shaped by structural and biological factors that disproportionately affect higher-BMI populations. Addressing this inequity requires proportionate resource allocation: directing additional nutritional, exercise, and ophthalmic screening resources to overweight and obese subgroups. This operationalizes the principle of proportionate universalism—providing universal access to baseline health promotion while disproportionately targeting resources to those with the greatest need. Achieving health equity in myopia prevention requires not only promoting physical fitness for all but also ensuring that its benefits are accessible to all body size groups—a principle that has yet to be operationalised in current public health practice.

### 4.4 Study limitations and future research directions

Several methodological limitations must be acknowledged to ensure rigorous interpretation of the present findings. First, the cross-sectional design precludes causal inference; the observed moderating association reflects population-level correlational patterns rather than directional causal effects. Longitudinal cohort studies or controlled intervention trials are required in subsequent research to validate the long-term dynamic moderating impact of BMI on fitness-myopia trajectories. Nevertheless, cross-sectional designs remain valuable for hypothesis generation and effect modification identification in population health research, particularly when the research question concerns differential subgroup responsiveness rather than causal mechanisms. Our identification of a substantial, graded interaction (*β* = −0.009, *p* < 0.001) across a well-characterized sample, together with a clear descending gradient in stratified analyses (0.088 → 0.014 → –0.035) and a Johnson-Neyman threshold (BMI_c = 0.744, corresponding to raw BMI ≈ 26.45 kg/m^2^), provides a robust empirical basis for designing future intervention trials and refining existing population health programmes. Notably, post-hoc power analysis revealed that the interaction effect was detected with approximately 97% power (*f^2^* = 0.051, Δ*R^2^* = 0.045, *N* = 300, *α* = 0.05), for the overall interaction effect in the full sample, supporting the reliable detection of the BMI-fitness moderating association across the entire study population. However, adequate global power does not guarantee stable subgroup estimation. Given the extremely limited number of participants with both high fitness and obesity in our sample (only 1% of the obese group achieved an “excellent” fitness rating), the interpretation of the moderating pattern within the obese stratum remains constrained by cell sparsity and should be treated with caution.

Second, this study draws on existing routine campus physical examination datasets, which lacked information on key unmeasured confounders including daily outdoor duration, near-work load, parental myopia history, and long-term dietary patterns. In the main regression models, we adjusted for sex; axial length was included only as a sensitivity analysis (Supplementary Table S1) rather than as a primary covariate, given its role on the causal pathway between physical fitness and refractive status. Residual confounding from unmeasured behavioral factors cannot be entirely ruled out. Moreover, without direct measures of outdoor time and near-work intensity, we cannot fully attribute the observed fitness-myopia association to outdoor light exposure; academic burden and physical fitness may also be entangled in ways that could influence both BMI and myopia risk. Nevertheless, even in these simplified models without behavioral covariates, the fitness × BMI interaction remained highly significant (*p* < 0.001), and the stratified regression showed a clear descending gradient (0.088 → 0.014 → –0.035), suggesting that the moderating effect is robust to unmeasured behavioral confounders.

Third, participants were recruited from a single comprehensive university in eastern China; this single-centre sampling framework limits the nationwide generalizability of the stratified threshold and moderating effect estimates. Multi-centre population surveys covering universities across diverse regional economic and educational contexts are warranted to replicate and extend the present results.

With respect to future research agendas aligned with population health priorities, subsequent work could further explore the socioeconomic disparities underlying the co-occurrence of low fitness, high BMI, and severe myopia, and evaluate the cost-effectiveness of stratified integrated intervention strategies. Additional research could also examine whether the moderating threshold of BMI (approximately 26.45 kg/m^2^, as identified by the Johnson-Neyman analysis) varies across different age groups, academic majors, and residential environments, to refine precision public health prevention schemes for youth visual health. Finally, qualitative research exploring the barriers and facilitators to physical activity participation among overweight and obese university students would complement our quantitative findings and inform the design of more acceptable and effective interventions.

## 5 Conclusion

This cross-sectional study examined the moderating role of BMI in the relationship between physical fitness and myopia among Chinese university students. Three main conclusions can be drawn from our findings.

First, physical fitness was independently associated with a more favorable refractive status in this population, suggesting that regular physical activity may confer tangible benefits to young adults‘visual health. Second, this protective association is significantly attenuated among individuals with higher BMI, as evidenced by a significant negative interaction between fitness and BMI (*β* = −0.009, CI: −0.013 to −0.004, *p* < 0.001) and a graded decline in protection from low to high BMI groups. Third, pronounced visual health disparities exist across weight subgroups: normal-weight students maintain better refractive status and higher fitness levels, whereas overweight and obese students bear compound burdens of poor fitness and more severe myopia.

From a public health perspective, these findings challenge the assumption that uniform physical activity recommendations confer equal benefits across all population subgroups. The identification of differential responsiveness to physical activity by BMI status—whereby higher-BMI individuals derive diminished myopia protection from fitness—highlights an often-overlooked equity gap in current youth health promotion strategies. We recommend that public health practitioners and policymakers adopt stratified intervention frameworks that: (1) integrate fitness, BMI, and vision screening data for risk stratification; (2) implement proportionate universalism—providing enhanced, multi-component interventions for overweight and obese subgroups; and (3) monitor equity indicators to ensure that health improvements are distributed across all body size groups.

Several limitations should be acknowledged, including the cross-sectional design, unmeasured confounders, and the single-centre sample. Despite these, this study provides novel evidence that BMI moderates the fitness–myopia association and offers an empirically grounded basis for designing precision public health strategies that promote equitable vision health outcomes across diverse student populations.

## Data Availability

The original contributions presented in the study are included in the article/Supplementary material, further inquiries can be directed to the corresponding author.
