# Supplementary material for: BMI moderates the association between physical fitness and spherical equivalent in Chinese college students: a cross-sectional study
Source: Front Public Health. 2026 Sep 24;14:1937229. doi: 10.3389/fpubh.2026.1937229 (PMC13646059; doi:10.3389/fpubh.2026.1937229)
Supplement: Supplementary file 1 [file Data_Sheet_1.PDF]

Supplementary Table S1. Multiple Linear Regression Analysis Predicting Spherical Equivalent

Variable	Model 1 (Main effects)		Model 2 (Interaction)	
	**β (95% CI)**	**p-value**	**β (95% CI)**	**p-value**
**Physical fitness (centered)**	0.025 (0.914, 4.132)	**0.001**	0.023 (0.008, 0.037)	**0.003**
**BMI (centered)**	0.023 (–0.024, 0.703)	0.333	0.016 (–0.031, 0.064)	0.498
**Physical fitness × BMI**	—	—	–0.003 (–0.006, –0.000)	**0.046**
**Axial length (mm)**	–1.629 (–1.765, –1.493)	**<0.001**	–1.603 (–1.740, –1.465)	**<0.001**
**Sex (ref: male)**	–1.637 (–1.990, –1.283)	**<0.001**	–1.555 (–1.916, –1.194)	**<0.001**
**Constant**	38.412 (34.909, 41.915)	**<0.001**	37.595 (34.019, 41.172)	**<0.001**
**Model fit**				
R^2	0.680		0.684	
Adjusted R^2	0.676		0.679	
ΔR^2 (vs. Model 1)	—		0.004	
F-statistic (df)	156.72 (4, 295)	**<0.001**	127.46 (5, 294)	**<0.001**
Likelihood-ratio test	—		$\chi^2 = 4.06$, p = 0.044	

Note. Unstandardized coefficients reported. Physical fitness and BMI were mean- centered to reduce multicollinearity; interaction term from centered variables. SE = spherical equivalent, AL = axial length. Sex: 1 = male, 2 = female.

Supplementary Table S2. Stratified regression analysis of physical fitness on spherical equivalent (SE) by BMI categories

BMI category	n	β (95% CI)	p-value	R^2
Normal weight	100	0.088 (0.047, 0.130)	< 0.001	0.159
Overweight	100	0.014 (–0.024, 0.053)	0.455	0.246
Obese	100	–0.035 (–0.086, 0.015)	0.170	0.024

Note. All models were adjusted for sex. β represents the unstandardized coefficient of physical fitness on SE (D per 1–unit increase in fitness score). Values in parentheses are 95% confidence intervals. Statistically significant results ($p < 0.05$) are indicated in bold.
